# Equine infectious anemia virus blocks interferon responses through Rev-mediated activation of the stress granule-PKR-eIF2α pathway

**DOI:** 10.1371/journal.ppat.1014262

**Published:** 2026-05-21

**Authors:** Huiling Ren, Kewei Chen, Bingqian Zhou, Weiguo Zhang, Xue-Feng Wang, Xiaojun Wang

**Affiliations:** 1 State Key Laboratory of Animal Disease Control and Prevention, Harbin Veterinary Research Institute, Chinese Academy of Agricultural Sciences, Harbin, Heilongjiang, China; 2 China-Kazakhstan Joint Laboratory for Herbivorous Animal Disease Research, Harbin Veterinary Research Institute, Chinese Academy of Agricultural Sciences, Harbin, Heilongjiang, China; 3 Institute of Western Agriculture, Chinese Academy of Agricultural Sciences, Changji, China; Sun Yat-Sen University, CHINA

## Abstract

Type I interferons (IFNs) play a pivotal role in antiviral defence by inducing interferon-stimulated genes (ISGs) that target multiple stages of viral replication. Equine infectious anemia virus (EIAV) is an ancient lentivirus that establishes long-term asymptomatic infections in equids, suggesting its exceptional immune evasion capabilities. However, the mechanisms by which EIAV modulates IFN responses remain unclear. Here, we demonstrate that EIAV infection attenuates ISG expression at the post-transcriptional level. The viral Rev protein plays a central role by interacting with the stress granule (SG) nucleating protein Ras-GTPase-activating protein binding protein 1 (G3BP1) to induce SG formation. This triggers the phosphorylation of double-stranded RNA-dependent protein kinase (PKR) and eukaryotic initiation factor 2α (eIF2α), which then block the translation of ISGs. Knockdown of G3BP1 or inhibition of PKR activation restores ISGs expression and enhances the antiviral effect of IFN against EIAV. Furthermore, the dimerization and RNA-binding domains of Rev are essential for SG assembly and the subsequent inhibition of IFN responses. Collectively, our findings reveal that EIAV has evolved a unique strategy to evade IFN responses, where the Rev protein reprograms SGs into proviral platforms by suppressing the translation of ISGs.

## Author summary

ISGs impose critical barriers to lentiviral replication through diverse antiviral mechanisms. Previous studies have found that lentiviruses, including HIV-1 and EIAV, utilize distinct proteins to specifically target and block different proteins encoded by ISGs. Here, we report a broad-spectrum inhibitory mechanism against ISG expression induced by EIAV Rev protein through modulation of the cellular stress responses to suppress protein synthesis. Mechanistically, Rev actively induces SG assembly through its interaction with G3BP1, subsequently activating the PKR-eIF2α pathway. Remarkably, this process subverts the innate antiviral function of the SG and is beneficial for virus replication. Our study reveals a unique immune evasion paradigm mediated by EIAV Rev protein, and provides novel insights into how viruses evade host immune responses and establish infections.

## Introduction

Equine Infectious Anemia Virus (EIAV) is a lentivirus that primarily infects equids, including horses, donkeys, and mules [1]. It causes Equine Infectious Anemia (EIA), a chronic and potentially fatal disease characterized by recurrent fever, anemia, and long-term immunosuppression. EIA is of significant global veterinary concern, with outbreaks leading to substantial economic losses in the equine industry due to the culling of infected animals and trade restrictions [2]. The EIAV genome is approximately 8.0 kb in length. It encodes several structural proteins, including Gag, Pol, and Env, as well as a number of accessory proteins such as Tat, Rev, Mat, Grev, and S2 [3]. Unlike hosts infected with other lentiviruses, most EIAV-infected horses become lifelong inapparent carriers by eliciting immune control over virus replication. This distinctive capacity for immunological containment means that EIAV offers a valuable model system to study host-pathogen coevolution and mechanisms of chronic viral control.

Type I interferons (IFNs) constitute the first line of the innate immune response against viral infections, including EIAV [4]. Upon viral infection, pattern recognition receptors recognize viral pathogen-associated molecular patterns and initiate a signaling cascade that culminates in the production of IFNs [5]. These secreted IFNs elicit activation of the Janus kinase-signal transducer and activator of transcription (JAK-STAT) pathway, leading to the transcription of hundreds of interferon-stimulated genes (ISGs) [5–7]. These ISGs encode proteins that restrict viral replication at multiple stages of the viral life cycle. For example, tetherin blocks viral particle release, SAM and HD domain containing deoxynucleoside triphosphate triphosphohydrolase 1 (SAMHD1) restricts reverse transcription by depleting dNTP pools, interferon-induced transmembrane protein 3 (IFITM3) inhibits viral membrane fusion, anti-melanoma differentiation-associated protein 5 (MDA5) enhances viral RNA sensing to amplify IFN production, and interferon-stimulated gene 15 (ISG15) disrupts viral release through ISGylation of host tumor susceptibility gene 101 protein [8–12]. IFNs also play a crucial role in bridging innate and adaptive immunity by enhancing antigen presentation and activating immune cells such as natural killer cells and T cells [5,13].

EIAV infection has been shown to elevate serum levels of IFN-α, which are inversely correlated with platelet counts and are considered contributing factors to EIAV-associated thrombocytopenia [14]. In addition, EIAV infection triggers the expression of type I IFNs in equine monocyte-derived macrophages (eMDMs), and the magnitude of this response correlates with the protective efficacy of attenuated vaccine strains [15]. Concurrently, the mRNA levels of several ISGs, such as Viperin and SLFN11, are elevated upon EIAV infection [16,17]. However, whether this upregulation translates to increased protein expression has not yet been determined. Despite this pronounced ISG induction at the transcriptional level, EIAV establishes persistent infection in the majority of infected horses, suggesting that the virus has evolved effective countermeasures to evade IFN-mediated antiviral responses. Indeed, EIAV deploys specific strategies to antagonize select ISG-mediated restrictions, such as utilizing its Rev protein to degrade SAMHD1, and its Env and S4 proteins to antagonize tetherin [18–20]. However, these targeted strategies cannot fully explain the unique capacity of EIAV for asymptomatic persistence, suggesting the existence of further mechanisms of ISG suppression. How EIAV modulates IFN signaling and evades antiviral responses remains largely unknown.

In this study, we found that EIAV infection attenuates the induction of ISGs post-transcriptionally. Specifically, EIAV infection induces a global shutoff of host protein synthesis, which is mediated by the phosphorylation of eukaryotic initiation factor 2α (eIF2α) through the double-stranded RNA-dependent protein kinase (PKR). Further investigation revealed that the viral Rev protein plays a central role in this process. Rev interacts with Ras-GTPase-activating protein binding protein 1 (G3BP1), a key component of stress granules (SGs), to induce SG formation. This SG formation triggers PKR activation and eIF2α phosphorylation, resulting in the suppression of ISG translation. Importantly, knockdown of G3BP1 or inhibition of PKR activation restores ISG protein expression and enhances the antiviral effect of IFN against EIAV. We also identified the dimerization and RNA-binding domains of Rev as being crucial for its ability to promote SG assembly and block interferon responses. Notably, this mechanism is specific to EIAV Rev, as HIV-1 Rev neither interacts with G3BP1 nor induces SG formation. This suggests a specialized adaptation of EIAV that may not be broadly conserved among other lentiviruses. Thus, we provide evidence that EIAV manipulates the Rev-SG-PKR-eIF2α axis to facilitate viral replication and evade host innate immunity. Taken together, this study identifies a novel function of the EIAV Rev protein in immune evasion, and unveils a unique viral strategy in which EIAV co-opts the SG to suppress host antiviral defenses.

## Results

### EIAV infection attenuates induction of ISG protein

**Fig 1:**
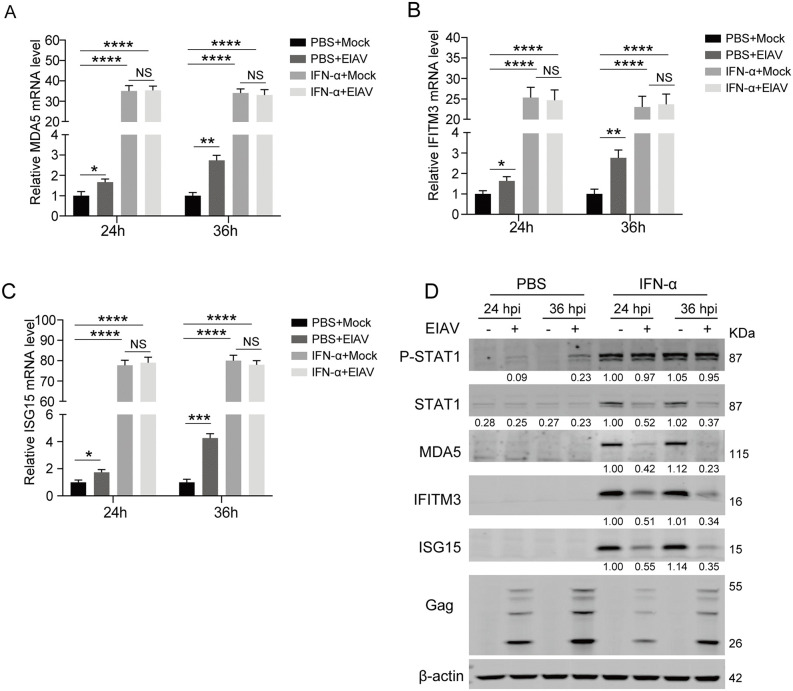
EIAV infection attenuates induction of ISG protein. (**A-C**) mRNA levels of MDA5, IFITM3, and ISG15 in uninfected and EIAV-infected eMDMs. eMDMs were either mock-infected or infected with EIAV (MOI = 1) for 12 or 24 h, and then treated with either 100 U/mL IFN-α or mock-treated with phosphate-buffered saline (PBS) for an additional 12 hours. Cellular RNA was extracted, and the levels of MDA5 **(A)**, IFITM3 **(B)**, and ISG15 (C) mRNA were determined using RT-qPCR. β-actin mRNA quantification from the same samples was used for normalization. Data represent means ± SD of three independent experiments. **(D)** Protein levels of MDA5, IFITM3, and ISG15 in uninfected and EIAV-infected eMDMs. Western blot analysis was performed to examine the protein expression of STAT1, p-STAT1, MDA5, IFITM3, and ISG15 in the samples corresponding to Fig 1A. β-actin was used as a loading control, and EIAV infection was verified using an anti-P26 antibody. The intensities of the protein bands were analyzed using the Odyssey Imaging System to calculate values relative to those of β-actin. Results were normalized to IFN-α-treated mock cells (24 h) as the control group, which was set to 1.

To investigate whether EIAV modulates interferon signaling, we first examined the expression of three classical ISGs (MDA5, IFITM3, and ISG15) in both infected and uninfected eMDMs, the natural target cells for viral replication. eMDMs were infected with EIAV at a multiplicity of infection (MOI) of 1 for either 24 or 36 hours. As shown in Fig 1A-1C, EIAV infection induced a modest upregulation of ISG mRNA levels in eMDMs, with fold changes ranging from 1.6 to 4.3. However, western blot analysis revealed no corresponding increase in ISG protein expression (Fig 1D, lanes 1–4), suggesting a post-transcriptional suppression. To determine whether this suppression results from impaired JAK-STAT signaling, we examined STAT1 phosphorylation following EIAV infection. Phosphorylation of STAT1 is a critical event downstream of type I IFN receptor engagement and a key readout of pathway activation [6,7]. Notably, EIAV infection alone significantly upregulated phosphorylated STAT1 levels without affecting total STAT1 expression (Fig 1D, lanes 1–4), indicating that EIAV triggers STAT1 activation.

To further dissect the relationship between IFN signaling and EIAV-induced translational suppression, we next assessed the response of infected cells to exogenous type I IFN stimulation. eMDMs were infected with EIAV at a multiplicity of infection (MOI) of 1 for either 12 or 24 hours. Subsequently, the cells were stimulated with IFN-α or IFN-β for an additional 12 hours before sample collection. Both IFN-α and IFN-β treatment triggered robust STAT1 phosphorylation in infected and uninfected cells at 24 and 36 hours post-infection (hpi), with no significant difference between the two groups (Fig 1D, lanes 5–8; S1D Fig, lanes 5–8), indicating that EIAV does not impair proximal JAK-STAT signaling. Consistently, IFN-α treatment triggered comparable upregulation of ISG transcripts in both experimental groups, with infected and uninfected cells showing similarly robust mRNA induction (23- to 81-fold increase). In contrast, western blot analysis revealed significantly attenuated ISG protein expression in IFN-α-treated EIAV-infected cells compared to IFN-α-treated uninfected controls (Fig 1D, lanes 5–8). Notably, a similar reduction in protein accumulation was observed for STAT1, which is also an ISG. Similarly, IFN-β stimulation yielded comparable results under the same experimental conditions, albeit with slight differences in induction magnitude (S1A-E Fig). Collectively, these results demonstrate that EIAV infection attenuates the induction of ISG proteins post-transcriptionally in infected eMDMs, and this suppression is not mediated by disrupting IFN-induced JAK1-STAT1 phosphorylation.

### EIAV suppresses ISG protein production through the PKR-eIF2α pathway

**Fig 2:**
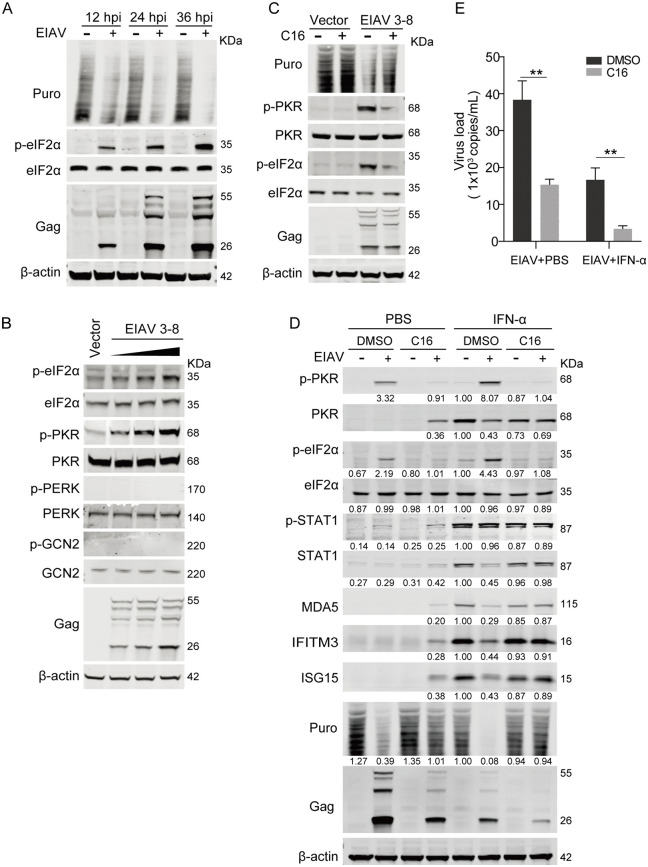
EIAV suppresses ISG protein production through the PKR-eIF2α pathway. (A) Assessment of host translation activity in EIAV-infected eMDMs using a ribopuromycylation assay. eMDMs were infected with EIAV at an MOI of 1 for 12, 24, or 36 hours. Prior to harvest, cells were pulsed with 5 µg/mL puromycin for 30 min to label newly synthesized proteins. Whole cell lysates were analyzed using western blotting with the indicated antibodies. (B) Dose-dependent activation of the PKR-eIF2α pathway resulting from EIAV proviral DNA expression. HEK293T cells were seeded into 6-well plates and transfected with increasing concentrations (250, 500, and 1000 ng) of pCMV3-8 or empty vector. At 24 hpt, whole cell lysates were analyzed using western blotting with the indicated antibodies. (C) Treatment with the PKR inhibitor C16 blocks activation of the PKR-eIF2α pathway resulting from EIAV proviral DNA expression. HEK293T cells were transfected with pCMV3-8 (1 μg) or empty vector. At 16 hpt, cells were treated with 1 μM C16 or DMSO for an additional 12 h before harvest. Prior to harvest, cells were pulsed with 5 µg/mL puromycin for 30 min to label newly synthesized proteins. Whole cell lysates were analyzed using western blotting with the indicated antibodies. (D) Treatment with the PKR inhibitor C16 restores expression of ISG proteins in EIAV-infected cells. eMDMs were infected with EIAV (MOI = 1) for 10 h, then treated with 1 μM C16 or DMSO for 2 h prior to stimulation with 100 U/mL IFN-α or PBS for 12 h. Prior to harvest, cells were pulsed with 5 µg/mL puromycin for 30 min to label newly synthesized proteins. Whole cell lysates were analyzed using western blotting with the indicated antibodies. The intensities of the protein bands were analyzed to calculate values relative to those of β-actin. Results were normalized to IFN-α-treated mock cells that were treated with DMSO. (E) C16 treatment reduces viral replication. Cell culture supernatants from samples corresponding to those in Fig 2D were collected. Viral RNA was extracted from the supernatants and quantified using qPCR to determine viral copy numbers. Data represent means ± SD of three independent experiments.

The persistence of ISG transcripts alongside reduced protein levels suggests translational inhibition by EIAV. To test that hypothesis, we examined the impact of EIAV infection on protein synthesis using a ribopuromycylation assay. EIAV infection induced a progressive, viral load-dependent suppression of global host protein synthesis (Fig 2A). Transfection with EIAV infectious clone plasmid (pCMV3–8) similarly resulted in a dose-dependent reduction in global protein synthesis (S2A Fig). Notably, HEK293T cells co-transfected with increasing doses of pCMV3–8 and pRL-TK, a Renilla luciferase reporter plasmid that allows rapid and sensitive quantification of protein production by measuring Renilla luciferase activity, showed a dose-dependent reduction in Renilla luciferase activity (S2B Fig), despite having stable mRNA levels (S2C Fig). Similarly, co-expression of pCMV3–8 with pEGFP-N1, an EGFP reporter plasmid that enables direct visualization and quantification of protein production via fluorescence intensity, resulted in significantly reduced fluorescence intensity (2.9-fold decrease; S2D and S2E Fig), despite no significant differences being detected in EGFP mRNA levels (S2F Fig). This reduction was further quantified by flow cytometry, revealing a 3.4-fold decrease in mean fluorescence intensity (MFI) of EGFP signals (S2G and S2H Fig). These findings collectively demonstrate that EIAV induces host protein synthesis shutoff through a transcription-independent mechanism.

eIF2α serves as a central regulator of translation initiation, with its phosphorylation status determining global protein synthesis rates during various stress conditions, including viral infection [21,22]. Given the inhibition of protein synthesis during EIAV infection, we examined the phosphorylation status of eIF2α. As shown in Fig 2A, EIAV infection specifically induced eIF2α phosphorylation, which suppressed host translation while allowing viral protein synthesis to continue. This suggests that EIAV may inhibit host protein synthesis by activating the eIF2α phosphorylation pathway and that viral protein synthesis is relatively resistant to the inhibitory effects of phosphorylated eIF2α. The phosphorylation of eIF2α is regulated by four kinases: general control nonderepressible-2 (GCN2), protein kinase R-like endoplasmic reticulum kinase (PERK), PKR, and heme-regulated inhibitor (HRI) [21,22]. To identify the kinase responsible for eIF2α phosphorylation, HEK293T cells were transfected with increasing concentrations of pCMV3–8. Western blot analysis demonstrated a dose-dependent increase in PKR phosphorylation (Fig 2B), but had no effect on the phosphorylation of GCN2 or PERK. To investigate the role of PKR in EIAV-mediated eIF2α phosphorylation, the PKR inhibitor C16 was utilized to inhibit the function of PKR. As shown in Fig 2C, C16 treatment effectively abolished the phosphorylation of both PKR and eIF2α, confirming that PKR serves as the primary kinase responsible for eIF2α phosphorylation in EIAV-infected cells. Importantly, this pharmacological inhibition of PKR also restored global protein synthesis, as measured by puromycin incorporation, indicating that PKR-mediated eIF2α phosphorylation is responsible for the translational suppression during EIAV infection.

Subsequently, we investigated the impact of PKR inhibition on the IFN response during EIAV infection. eMDMs were infected with EIAV for 10 hours and then treated with C16 for 2 hours prior to IFN-α stimulation. Protein extracts were analyzed using western blotting to examine ISG protein levels, PKR and eIF2α phosphorylation. As shown in Fig 2D, inhibition of PKR activity restored ISG protein expression in EIAV-infected cells with or without IFN-α stimulation. Notably, IFN-α treatment robustly induced PKR protein accumulation in uninfected eMDMs, consistent with its identity as a canonical ISG. However, this induction was markedly suppressed in EIAV-infected cells, displaying a similar expression pattern to that of MDA5, IFITM3, and ISG15. Remarkably, despite reduced PKR protein levels, EIAV infection significantly enhanced IFN-induced PKR phosphorylation and subsequent eIF2α activation compared to uninfected controls (Fig 2D). Consistently, global protein synthesis in EIAV-infected cells was also restored upon C16 treatment under both unstimulated and IFN-α-stimulated conditions. Moreover, pharmacological inhibition of PKR activity resulted in significant suppression of viral replication, as evidenced by a 2.7-fold decrease in viral RNA copy numbers compared to those of the DMSO-treated controls (Fig 2E). This antiviral effect was further amplified in IFN-α-treated cells, where C16 synergized with IFN-α to achieve a 4.9-fold greater reduction in viral RNA copy numbers compared to IFN-α treatment alone (Fig 2E). These data collectively demonstrate that EIAV-induced PKR-eIF2α activation plays a pivotal role in attenuating the IFN response through suppression of the translation of ISGs.

### EIAV Rev activates the PKR-eIF2α pathway to suppress induction of ISG protein

**Fig 3:**
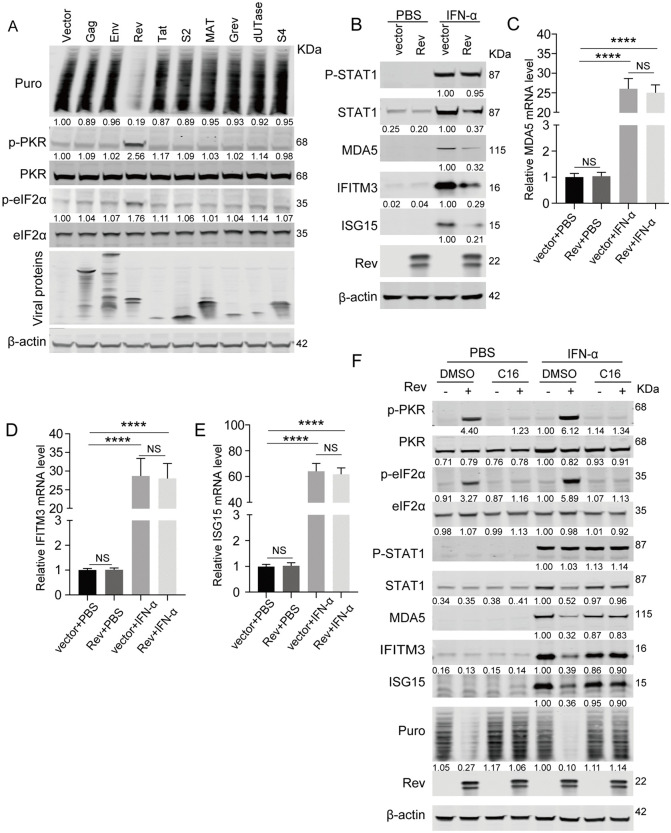
EIAV Rev activates the PKR-eIF2α pathway to suppress induction of ISG protein. (A) Screening of viral proteins for inducing phosphorylation of PKR and eIF2α. HEK293T cells were transfected with HA-tagged Gag, Env, Tat, Rev, S2, Mat, Grev, dUTPase, S4 or with empty vector. At 24 hpt, cells were pulsed with 5 µg/mL puromycin for 30 min to label newly synthesized proteins prior to harvest. Protein expression was analyzed using western blotting with the indicated antibodies. The intensities of the protein bands were analyzed to calculate values relative to those of β-actin. Results were normalized to control cells. (B) Overexpression of Rev inhibits expression of interferon-stimulated proteins. HEK293T cells were transfected with pcDNA 3.1-Rev-HA or empty vector (500 ng). After 20 h, cells were stimulated with 100 U/mL IFN-α or PBS for 12 h. Prior to harvest, cells were pulsed with 5 µg/mL puromycin for 30 min to label newly synthesized proteins. Protein expression was analyzed using western blotting with the indicated antibodies. The intensities of the protein bands were analyzed to calculate values relative to those of β-actin. Results were normalized to IFN-α-treated cells transfected with empty vector. (C-E) Overexpression of Rev does not affect ISG mRNA levels. RT-qPCR analysis was performed to examine the levels of MDA5 (C), IFITM3 (D), and ISG15 (E) mRNA in the samples corresponding to those in Fig 3B. Data represent means ± SD of three independent experiments. (F) Treatment with the PKR inhibitor C16 restores expression of ISG protein suppressed by Rev overexpression. HEK293T cells were transfected with Rev-expressing plasmid for 16 hours and then treated with the PKR-specific inhibitor C16 (0.8 μM) for 2 hours prior to IFN-α stimulation (100 IU/mL) for 12 hours. Prior to harvest, cells were pulsed with 5 µg/mL puromycin for 30 min to label newly synthesized proteins. Protein expression was analyzed using western blotting with the indicated antibodies. The intensities of the protein bands were analyzed to calculate values relative to those of β-actin. Results were normalized to IFN-α-treated cells transfected with empty vector (DMSO control).

To identify the viral proteins responsible for PKR activation during EIAV infection, HEK293T cells were transfected with expression vectors carrying EIAV *gag*, *env*, *ta*t, *rev*, *s2*, *mat*, *grev*, *dUTPase*, or the *s4* gene. As shown in Fig 3A, Rev was the most potent activator of the PKR-eIF2α pathway, and treatment was accompanied by significant suppression of global protein synthesis. Furthermore, EIAV Rev expression reduced global protein synthesis while increasing PKR and eIF2α phosphorylation in a dose-dependent manner (S3A Fig). When Rev was overexpressed, we observed a progressive decrease in Renilla luciferase activity (S3B Fig) without affecting the corresponding mRNA levels (S3C Fig). These results were further corroborated using immunofluorescence analysis, which showed markedly reduced puromycin incorporation in Rev-expressing cells compared to the controls (S3D Fig).

To investigate the role of Rev in modulating interferon signaling, HEK293T cells were transfected with Rev-expressing plasmids for 20 hours prior to IFN-α treatment. Our results demonstrated that Rev overexpression significantly suppressed IFN-induced protein expression, including that of STAT1, MDA5, IFITM3, and ISG15 (Fig 3B). Notably, Rev overexpression had no effect on IFN-induced STAT1 phosphorylation, indicating that the observed suppression occurs downstream of STAT1 activation. Interestingly, despite this inhibition at the protein level, IFN-α stimulation induced comparable mRNA levels of these ISGs in both Rev-expressing and control cells (Fig 3C-3E). To examine the involvement of PKR in this process, HEK293T cells were transfected with Rev-expressing plasmids for 16 hours, then treated with the PKR inhibitor C16 for 2 hours prior to IFN-α stimulation. As shown in Fig 3F, C16 treatment effectively blocked Rev-induced PKR and eIF2α phosphorylation. Consistently, global protein synthesis in Rev-expressing cells was restored upon C16 treatment, indicating that Rev suppresses translation in a PKR-dependent manner. Furthermore, C16 treatment restored IFN-induced protein expression in Rev-expressing cells, while having no significant effect on basal ISG levels in unstimulated cells. Although IFN stimulation caused only a modest reduction in total PKR protein levels in Rev-transfected cells compared to the controls, it triggered markedly enhanced PKR and eIF2α phosphorylation. This paradoxical effect suggests that Rev protein selectively potentiates the activation of PKR molecules during interferon signaling. Together, these results demonstrate that Rev-induced PKR-eIF2α activation plays a critical role in impairing interferon responses through the inhibition of ISG translation.

### EIAV Rev triggers SG formation and colocalizes with G3BP1

**Fig 4:**
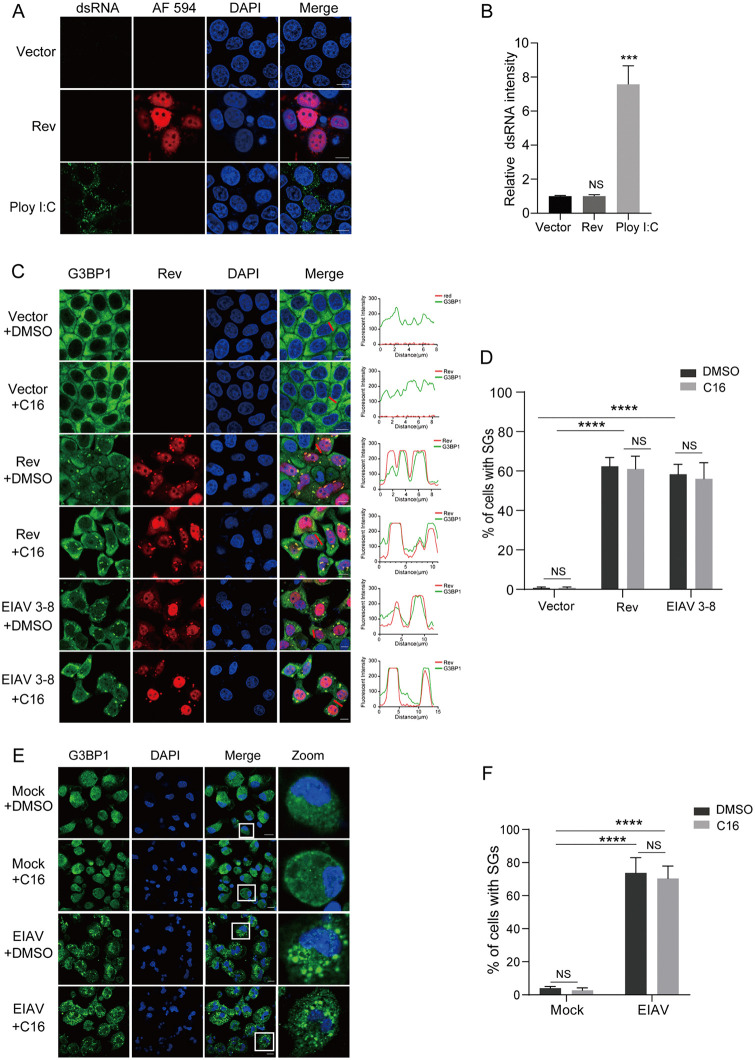
EIAV Rev triggers SG formation and colocalizes with G3BP1. **(A)** Rev expression does not induce dsRNA accumulation. HeLa cells were transfected with pcDNA 3.1-Rev-HA or pcDNA 3.1-HA (500 ng). As a positive control for dsRNA formation, a separate group of cells was transfected with poly(I:C) (1 μg/mL). At 24 hpt, cells were fixed and stained with the dsRNA-specific J2 antibody (green), anti-HA antibody (red, for Rev detection) and DAPI (blue) for nuclear staining. Scale bars: 10 µm. **(B)** Quantification of the relative dsRNA fluorescence intensity in Fig 4A. **(C)** Rev triggers SG formation. HeLa cells were transfected with pcDNA 3.1-Rev-HA, pCMV3-8, or pcDNA 3.1-HA (500 ng). At 16 hpt, cells were treated with 1 μM C16 or DMSO for an additional 8 h. Cells were fixed and stained with the anti-G3BP1 antibody (green), anti-Rev antibody (red) and DAPI (blue) for nuclear staining. Scale bars: 10 µm. Shown is an intensity profile of the linear region of interest (ROI) across the HeLa co-stained with G3BP1 and Rev. **(D)** Quantification of the SG-positive cells in Fig 4C. **(E)** EIAV infection induces SGs in eMDMs. eMDMs were infected with EIAV (MOI = 1). At 4 hpi, cells were treated with either 1 μM C16 or DMSO for 8 hours, then fixed and stained with an antibody specific to G3BP1 (green) and with DAPI (blue) for nuclear staining. Scale bars: 10 µm. **(F)** Quantification of the SG-positive cells in Fig 4E.

PKR is typically activated through binding to viral dsRNA intermediates formed during replication [23,24]. To elucidate the mechanism of Rev-mediated PKR activation, we first examined dsRNA production in Rev-expressing cells. Immunofluorescence analysis using the dsRNA-specific J2 antibody revealed no detectable dsRNA signals in cells transfected with Rev-expressing plasmids (Fig 4A and 4B), indicating a dsRNA-independent mechanism of PKR activation by Rev. Recent studies have identified SGs as important platforms for PKR activation [25–27]. SGs are dynamic cytoplasmic condensates containing translationally stalled mRNAs and RNA-binding proteins that assemble in response to various cellular stresses. These dynamic structures are characterized by the presence of marker proteins such as G3BP1, a tunable switch that triggers phase separation to assemble stress granules [28–30]. To investigate whether Rev triggers SG formation as a potential mechanism for PKR activation, we examined the formation of SGs in Rev-expressing cells. As shown in Fig 4C, Rev expression alone induced robust formation of G3BP1-positive punctate structures in the cytoplasm, with Rev showing pronounced colocalization with G3BP1 within these puncta. Consistently, cells transfected with pCMV3–8 also exhibited similar cytoplasmic puncta, where viral Rev displayed marked colocalization with G3BP1. To confirm that these G3BP1-positive structures are *bona fide* SGs containing translationally arrested mRNA, we performed oligo(dT) fluorescence in situ hybridization (FISH) to visualize poly(A) mRNA. Notably, poly(A) mRNA was readily detected within the Rev-positive granules in Rev-expressing cells and in cells transfected with the EIAV infectious clone (S4A Fig), confirming that the G3BP1 foci induced by EIAV and Rev are indeed functional SGs with entrapped mRNA. Moreover, EIAV infection triggered robust SG assembly, as evidenced by 63–81% of infected eMDMs containing G3BP1-positive granules at 12 hpi (Fig 4E).

We next investigated whether SG formation requires PKR activation. To test this, cells were transfected with Rev or the EIAV infectious clone pCMV3–8. At 16 hpt, cells were treated with the PKR-specific inhibitor C16 (1 µM) or mock-treated with DMSO for an additional 8 hours. As shown in Fig 4C and 4D, C16 treatment did not block SG formation in Rev-expressing cells or in cells transfected with pCMV3–8. Similarly, C16 treatment failed to inhibit SG assembly in EIAV-infected eMDMs (Fig 4E and 4F). These findings suggest that EIAV infection and Rev expression induce SG formation independently of PKR activation, positioning SG assembly as an upstream event in the Rev-mediated PKR activation pathway.

### EIAV Rev activates the PKR-eIF2α pathway and suppresses induction of ISG protein in a G3BP1-dependent manner

**Fig 5:**
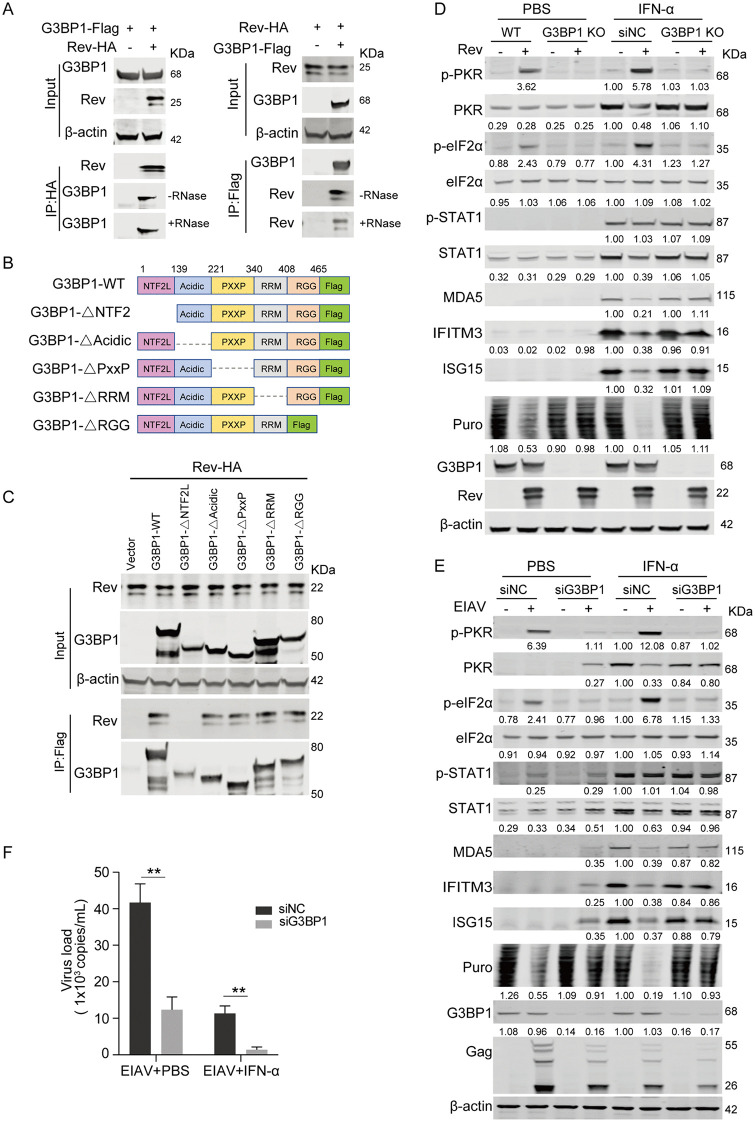
EIAV Rev activates the PKR-eIF2α pathway and suppresses induction of ISG protein in a G3BP1-dependent manner. (A) G3BP1 interacts with Rev. HEK293T cells were co-transfected with pcDNA3.1-Rev-HA (500 ng) and either an empty vector or pCMV-G3BP1-Flag (500 ng). At 24 hpt, cell lysates were prepared and treated with or without RNase A (100 μg/mL) before immunoprecipitation with anti-Flag M2 beads. Immunoprecipitates were then analyzed using western blotting with anti-HA, anti-Flag, and anti-β-actin antibodies. For reciprocal Co-IP, HEK293T cells were co-transfected with pCMV-G3BP1-Flag and either an empty vector or pcDNA3.1-Rev-HA. After 24 hpt, cell lysates were prepared and treated with or without RNase A before immunoprecipitation with anti-HA beads. The precipitated proteins were then analyzed using western blotting with the indicated antibodies. (B) Schematic diagram of wild-type G3BP1 and structures of respective mutants. (C) Rev Interacts with the NTF2L domain of G3BP1. HEK293T cells were co-transfected with pcDNA3.1-Rev-HA and either an empty vector, wild-type G3BP1, or truncated G3BP1-Flag. At 24 hpt, cells were harvested, immunoprecipitated with anti-Flag antibody, and further assessed using western blotting with the indicated antibodies. (D) Knockout of G3BP1 restores Rev-mediated suppression of ISG proteins. G3BP1 knockout HEK293T cells and wild-type controls were transfected with pcDNA3.1-Rev-HA or empty vector (500 ng) for 20 hours. Cells were then stimulated with 100 U/mL IFN-α for an additional 12 hours. Protein expression was analyzed using western blotting with the indicated antibodies. The intensities of the protein bands were analyzed to calculate values relative to those of β-actin. Results were normalized to IFN-α-treated wild-type cells transfected with empty vector. (E) Knockdown of G3BP1 restores expression of ISG proteins in EIAV-infected cells. eMDMs were transfected with G3BP1-specific siRNA for 24 hours, followed by infection with EIAV (MOI = 1) for 12 hours. Prior to harvest, cells were pulsed with 5 µg/mL puromycin for 30 min to label newly synthesized proteins. Cells were then stimulated with 100 U/mL IFN-α for an additional 12 hours. Protein expression was analyzed using western blotting with the indicated antibodies. The intensities of the protein bands were analyzed to calculate values relative to those of β-actin. Results were normalized to IFN-α-treated mock cells. (F) G3BP1 depletion inhibits viral replication. Cell culture supernatants from samples corresponding to Fig 5E were collected. Viral RNA was extracted from the supernatants and quantified with qPCR to determine viral copy numbers. Data represent means ± SD of three independent experiments.

The observation that Rev induces SG assembly and colocalizes with G3BP1 suggested a potential interaction between Rev and G3BP1. To test this, we performed co-immunoprecipitation (Co-IP) assays, and found that G3BP1 coimmunoprecipitated with Rev in an RNA-independent manner (Fig 5A). G3BP1 is composed of multiple functional domains, including an N-terminal nuclear transport factor 2-like domain (NTF2L), an acidic domain, a PXXP motif, an RNA recognition motif (RRM), and an arginine and glycine-rich region (RGG) at the C-terminus [31]. To identify the specific region of G3BP1 responsible for its interaction with Rev, we constructed a series of truncated G3BP1 mutants and examined their binding to Rev using Co-IP assays (Fig 5B). As shown in Fig 5C, Rev specifically interacts with the NTF2L domain of G3BP1, as deletion of this domain abolished the interaction.

G3BP1 is considered an SG nucleating protein, as its expression alone is sufficient to induce SG formation in the absence of exogenous stressors, and its depletion inhibits SG assembly in response to various stressors [32–34]. Given the critical role of G3BP1 in both SG assembly and PKR activation, we reasoned that Rev is likely to trigger SG formation and subsequent PKR phosphorylation through its interaction with G3BP1, thereby blocking the interferon response. To this end, G3BP1 knockout (KO) HEK293T cells were generated using the CRISPR-Cas9 system. Wild-type (WT) and G3BP1 KO HEK293T cells were transfected with a Rev-expressing plasmid for 20 hours, followed by stimulation with IFN-α for an additional 12 hours. Western blot analysis revealed that G3BP1 knockout attenuated Rev-induced phosphorylation of PKR and eIF2α (Fig 5D). Consistent with the reduction in eIF2α phosphorylation, G3BP1 deficiency rescued the Rev-mediated inhibition of global protein synthesis, as measured by puromycin incorporation. Moreover, in the presence of IFN-α stimulation, G3BP1 knockout restored the expression of ISGs, which were otherwise suppressed by Rev. IFN-α-induced STAT1 phosphorylation was not affected by G3BP1 knockout. The role of G3BP1 in Rev-mediated PKR activation was further assessed by overexpressing wild-type G3BP1 or a mutant lacking the NTF2L domain (S5A Fig). Consistent with previous studies, overexpression of wild-type G3BP1 alone induced PKR phosphorylation, whereas the NTF2L-deficient mutant did not. However, co-expression of Rev with either wild-type G3BP1 or the NTF2L mutant did not further enhance Rev-mediated PKR phosphorylation, indicating that endogenous G3BP1 is sufficient for Rev to activate PKR.

We next examined the role of G3BP1 in eMDMs during EIAV infection. Following 24 hours of G3BP1 knockdown using specific siRNA, eMDMs were infected with EIAV for 12 hours before being treated with IFN-α for an additional 12 hours. As shown in Fig 5E, G3BP1 depletion significantly restored ISG protein expression and reduced PKR and eIF2α phosphorylation, regardless of IFN-α stimulation. Consistent with the reduction in eIF2α phosphorylation, G3BP1 deficiency also rescued the EIAV-induced inhibition of global protein synthesis. IFN-α-induced STAT1 phosphorylation was not affected by G3BP1 knockdown. Importantly, the attenuation of PKR and eIF2α phosphorylation upon G3BP1 depletion correlated with diminished viral replication. G3BP1 knockdown reduced viral replication by 3.4-fold under basal conditions. This antiviral effect was significantly enhanced upon IFN-α stimulation, where G3BP1 depletion led to a more pronounced 8.1-fold reduction in viral replication compared to that in the control cells (Fig 5F). Collectively, these findings establish G3BP1 as a crucial host factor that facilitates Rev-mediated PKR activation and consequently the suppression of interferon-stimulated gene expression during viral infection.

Given the observed interaction between EIAV Rev and G3BP1, we next investigated whether HIV-1 Rev, the functional homolog from a different lentivirus, exhibits similar properties. To this end, cells were transfected with an HIV-1 Rev-expressing plasmid and assessed for G3BP1 interaction and SG formation. In contrast to EIAV Rev, Co-IP assays revealed that HIV-1 Rev did not associate with G3BP1 (S6A and S6B Fig). Furthermore, immunofluorescence analysis showed that HIV-1 Rev expression failed to induce the formation of G3BP1-positive SGs, remaining diffusely distributed throughout the cytoplasm without detectable colocalization with G3BP1 puncta (S6C and S6D Fig). These findings reveal that the capacity to interact with G3BP1 and induce SG formation is not a shared property between EIAV Rev and HIV-1 Rev.

### Identification of Rev domains crucial for the suppression of ISG protein induction

Lentiviral Rev proteins typically function to mediate nuclear export of unspliced viral mRNAs by binding the Rev responsive element (RRE) in viral pre-mRNA, thereby regulating viral gene expression [35]. EIAV Rev contains four domains: a bipartite RNA-binding domain consisting of central RRDRW and C-terminal KRRRK motifs, a nuclear export signal (NES), and a non-essential variable region (ND). The Leu residue at position 95 contributes to dimerization and is necessary for RNA-binding activity [36]. While the KRRRK motif was initially proposed as a nuclear localization signal, our prior work established its dispensability for nuclear targeting [18].

**Fig 6:**
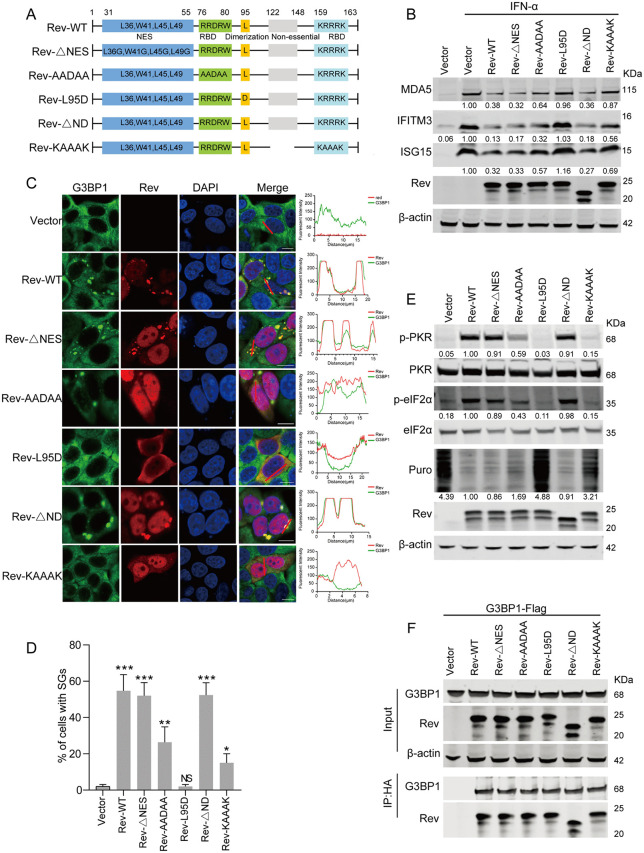
Identification of Rev domains crucial for the suppression of ISG protein induction. **(A)** Schematic diagram of wild-type EIAV Rev and structures of respective mutants. **(B)** Immunoblot analysis of ISG protein expression in HEK293T cells transfected with EIAV Rev mutants. HEK293T cells were transfected with plasmids encoding Rev-WT or with one of the five Rev mutants (500 ng) as described in Fig 6A. At 20 hpt, cells were stimulated with 100 U/mL IFN-α for an additional 12 hours. Cell lysates were then prepared and subjected to western blot analysis using the indicated antibodies. The intensities of the protein bands were analyzed to calculate values relative to those of β-actin. Results were normalized to IFN-α-treated cells transfected with wild-type Rev. **(C)** Immunofluorescence analysis of SG formation induced by Rev mutants. HeLa cells were transfected with plasmids encoding wild-type Rev or one of the various Rev mutants. At 24 hpt, cells were fixed and stained with the anti-G3BP1 antibody (green), anti-HA antibody (red, for Rev detection), and DAPI (blue) for nuclear staining. Scale bars: 10 µm. Shown is an intensity profile of the linear ROI across the HeLa co-stained with G3BP1 and Rev. **(D)** Quantification of SG-positive cells in Fig 6C. Data represent means ± SD of three independent experiments. **(E)** Immunoblot analysis of PKR/eIF2α phosphorylation and puromycin incorporation in HEK293T cells transfected with EIAV Rev mutants. HEK293T cells were transfected with plasmids encoding Rev-WT or with one of the five Rev mutants (500 ng) as described in Fig 6A. At 24 hpt, cells were pulsed with 5 µg/mL puromycin for 30 min to label newly synthesized proteins prior to harvest. Protein expression was analyzed using western blotting with the indicated antibodies. The intensities of the protein bands were analyzed to calculate values relative to those of β-actin. Results were normalized to cells transfected with wild-type Rev. **(F)** Co-IP analysis of G3BP1 and Rev mutants. HEK293T cells were co-transfected with pCMV-G3BP1-Flag and an empty vector, or wild-type Rev or one of the Rev mutants. At 24 hpt, cells were harvested, immunoprecipitated with anti-HA antibody, and further assessed using western blotting with the indicated antibodies.

To map the key domain of Rev responsible for the suppression of ISG protein expression, we constructed five EIAV Rev mutants, including a Rev NES mutant (Rev-ΔNES), two RNA binding domain mutants (Rev-AADAA and Rev-KAAAK), a dimerization domain mutant (Rev-L95D), and a non-essential domain mutant (Rev-ΔND) for the analysis (Fig 6A). As shown in Fig 6B, wild-type Rev effectively suppressed IFN-induced ISG upregulation, whereas the dimerization-deficient Rev-L95D mutant completely lost this inhibitory capacity, showing ISG levels comparable to empty vector controls. The RNA-binding mutants Rev-AADAA and Rev-KAAAK exhibited significantly attenuated suppression compared to wild-type Rev, retaining only partial inhibitory activity. Consistent with these findings, Rev-L95D failed to induce G3BP1 puncta formation, while Rev-AADAA and Rev-KAAAK exhibited significantly reduced percentages of cells containing G3BP1 granules compared to Rev-WT (Fig 6C and D). These granules were confirmed to contain mRNA by oligo(dT) FISH, confirming that the puncta induced by Rev variants represent *bona fide* stress granules (S7A Fig).

Importantly, these phenotypic differences aligned with their effects on the PKR-eIF2α pathway and subsequent protein synthesis. Puromycin incorporation assays revealed that Rev-L95D fully abrogated phosphorylation of PKR and maintained global translation, whereas the two RNA-binding mutants showed partial impairment of PKR phosphorylation, leading to reduced translational activity (Fig 6E). These results were further corroborated by immunofluorescence analysis of puromycin incorporation, which revealed varying degrees of reduced global translation in cells expressing Rev variants compared to controls (S7B Fig). In addition, all mutants maintained normal G3BP1-binding affinity, indicating that their suppression defects resulted from impaired RNA-binding and dimerization rather than compromised G3BP1 interaction (Fig 6F). This observation suggests that the reduced interferon suppression capacity of these mutants stems from deficiencies in RNA binding and dimerization, which are essential for SG formation, while their interaction with G3BP1 remains intact. In conclusion, the dimerization and RNA-binding capabilities of Rev are crucial for its ability to block interferon responses.

## Discussion

**Fig 7:**
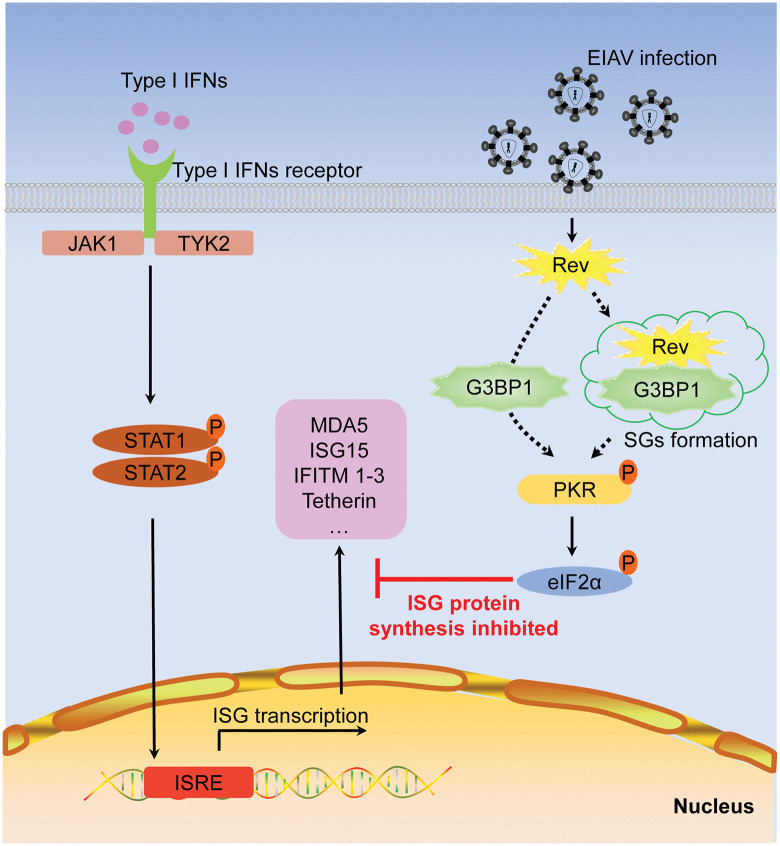
Schematic representation shows the model of EIAV-mediated suppression of interferon responses. The EIAV Rev protein interacts with G3BP1 to trigger SG formation and facilitate PKR activation. This Rev-mediated process triggers downstream eIF2α phosphorylation and inhibits the translation of ISGs, thereby facilitating viral immune evasion and promoting viral replication.

IFNs play a central role in antiviral defense through induction of ISGs that target multiple stages of the viral life cycle. EIAV stands out among lentiviruses by establishing asymptomatic persistence and being the only lentivirus for which an effective attenuated vaccine exists [37]. These unique characteristics make it an invaluable model for studying viral persistence and immune escape strategies, offering critical insights for developing novel antiviral interventions and informing vaccine development. We hypothesize that EIAV must employ additional mechanisms to downregulate ISG expression in order to replicate effectively in an IFN-rich environment, beyond simply targeting specific ISGs. Our research reveals a novel immune evasion mechanism employed by EIAV, which broadly downregulates ISG protein expression using its Rev protein. Specifically, Rev triggers SG formation and activates the PKR and eIF2α phosphorylation, thereby effectively blocking ISG translation to evade innate immune responses (Fig 7).

PKR is an interferon-induced serine/threonine kinase typically activated by viral dsRNA. Upon activation, PKR phosphorylates eIF2α at Ser51, leading to suppression of global protein synthesis [38]. Beyond its role in translation control, PKR enhances antiviral responses by promoting interferon induction [39,40]. This powerful antiviral mechanism has driven numerous viruses, such as HIV-1 and herpes simplex virus, to evolve sophisticated strategies for inhibiting PKR activation. HIV-1 employs multiple mechanisms to evade PKR-mediated antiviral effects. The viral Tat protein interacts with PKR, inhibiting its phosphorylation and downstream eIF2α activation [41]. Additionally, HIV-1 utilizes cellular factors, including TAR RNA binding protein and Adenosine Deaminase acting on RNA 1, which interact with PKR to suppress its phosphorylation, and repurposes the PKR activator from a PKR activator to an inhibitor in infected cells [42–44].

In contrast to HIV-1 Tat-mediated PKR inhibition, EIAV instead converts PKR activation into a proviral mechanism. Treatment with the PKR inhibitor C16 restored ISG protein expression in EIAV-infected cells both with and without IFN-α stimulation (Fig 2D). Furthermore, C16 treatment alone reduced viral replication by 2.7-fold, and this effect was enhanced to a 4.9-fold reduction when combined with IFN-α treatment (Fig 2E). These results strongly suggest that PKR-mediated host shutoff confers a critical survival advantage to EIAV during immune responses. EIAV and Hepatitis C virus (HCV) represent two rare examples that exploit PKR phosphorylation to evade immune responses, yet their mechanisms diverge substantially. EIAV induces rapid PKR activation through its Rev protein without requiring dsRNA, whereas HCV triggers delayed PKR phosphorylation (2 days post-infection) that is dependent on accumulated viral dsRNA [45–47]. We propose that the unique capacity of Rev to co-opt SG for PKR activation, distinct from the passive dsRNA-responsive mechanism employed by HCV, serves as the principal factor underlying the rapid immune evasion capability of EIAV and its subsequent establishment of persistent infection.

Phosphorylation of eIF2α triggers a host shutoff response that suppresses both host and viral protein synthesis, and which is commonly regarded as an innate defense mechanism against viral infection [21,22]. Intriguingly, despite eIF2α phosphorylation (Fig 2A), there is a paradoxical accumulation of viral proteins, indicating that EIAV has evolved translation mechanisms that circumvent canonical eIF2α-dependent pathways. Several viruses are known to have evolved sophisticated strategies to bypass eIF2α-mediated translation inhibition. HCV employs IRES-dependent translation initiation to recruit ribosomes independently of eIF2α phosphorylation [48,49]. Sindbis virus utilizes a downstream stem-loop in its subgenomic RNA to recruit 40S ribosomal subunits and initiate translation at non-AUG codons, thereby maintaining protein synthesis efficiency even under eIF2α phosphorylation [50,51]. The specific mechanism by which EIAV achieves viral protein production during translation shutoff remains to be determined.

We investigated the mechanism by which EIAV Rev mediates PKR phosphorylation, and found that EIAV Rev mediates PKR phosphorylation through a SG-dependent rather than dsRNA-dependent mechanism. Rev triggers PKR activation in the absence of detectable dsRNA, while robustly inducing SG assembly (Fig 4A-E). Importantly, Rev specifically interacts with the core SG component G3BP1, as demonstrated by both their strong colocalization in cytoplasmic foci and Co-IP assays (Fig 4C and 5A). The essential role of this interaction was confirmed by the abolition of Rev-induced PKR phosphorylation upon G3BP1 knockdown (Fig 5D). This observation is consistent with recent findings demonstrating that PKR can be activated within SGs independently of dsRNA [25,26,52]. SGs typically function as antiviral hubs by sequestering PKR and other innate immune sensors to amplify antiviral responses [53,54]. In response, numerous viruses have developed countermeasures against SG formation. For instance, HIV-1 effectively blocks SG assembly through the interaction between its Gag protein and host eukaryotic elongation factor 2, while picornaviruses encode proteases to cleave G3BP1 [34,55,56]. Contrary to the trend, EIAV Rev uniquely manipulates SG formation to promote infection, as evidenced by restored ISG expression and reduced viral replication following G3BP1 knockdown. The differences in SG modulation between HIV-1 and EIAV highlight the versatility of lentiviruses in adapting to their host environments. Notably, despite its functional homology to EIAV Rev in mediating viral RNA nuclear export, HIV-1 Rev failed to induce G3BP1-positive SG formation or interact with G3BP1 in Co-IP assays (S6 Fig). These observations indicate that the ability to engage SG machinery is not a conserved property among lentiviral Rev proteins, further underscoring the divergence in immune evasion strategies among lentiviruses.

In the assembly of SG, G3BP1 acts as a molecular switch that transitions from an auto-inhibited state to an open conformation upon RNA binding [57,58]. This conformational change enables multivalent interactions and facilitates the formation of condensates, thereby driving SG assembly. Whether SG formation functions upstream or downstream of PKR activation remains a matter of debate. Our data showing that C16 treatment does not block SG assembly (Fig 4) suggest a potential upstream role for SGs in this pathway. Although the data presented here do not directly prove that SG assembly is the causal trigger for PKR phosphorylation, previous studies have established that SGs are liquid-liquid phase separation condensates that serve as critical platforms for PKR activation [25–27]. Within these structures, the SG nucleating protein G3BP1 interacts with inactive PKR and recruits it to stress granules, where PKR is subsequently activated and released to phosphorylate eIF2α. Consistent with this platform model, we observe that Rev induces bona fide SG formation (Fig 4C and S4A Fig) and G3BP1 is essential for downstream PKR activation (Fig 5D-E). Nevertheless, an alternative possibility cannot be ruled out, namely that Rev activates PKR through an SG-independent pathway, in which the Rev-G3BP1 interaction directly induces PKR-eIF2α phosphorylation (Fig 7). However, definitive evidence would require uncoupling these events using eIF2α S51A mutant cell lines, which could determine whether SG formation can occur independently of eIF2α-mediated translation arrest. Despite multiple attempts using CRISPR-Cas9 editing, we were unable to generate viable homozygous eIF2α S51A mutant cell lines. We speculate that eIF2α phosphorylation at Ser51 may be essential for maintaining cellular homeostasis, and its complete abrogation likely confers a survival disadvantage, consistent with the established role of eIF2α in stress adaptation and cell viability [21,22].

Our results demonstrate that EIAV Rev interacts with the NTF2L domain of G3BP1 (Fig 5A-C) independently of the mRNA-binding or dimerization capabilities of Rev (Fig 6F). However, complete SG formation requires both of these additional Rev functions. We propose that Rev may act as a scaffold that crosslinks G3BP1 molecules through their NTF2L domains to drive their condensation. Furthermore, the mRNA-binding capability of Rev could facilitate this process by enriching specific mRNAs within the granules, creating a localized microenvironment that promotes SG formation. Given the essential function of Rev, it was not feasible to construct infectious clones containing functionally impaired Rev for evaluating its effects on interferon signaling and translation inhibition during viral infection. Future investigations should focus on identifying the critical residues pivotal solely for the G3BP1 interaction without compromising its capacity to facilitate the nuclear export of the Rev-mRNA complex.

In summary, our findings identify a novel role of EIAV Rev in evading host immune responses, where it is capable of mediating broad-spectrum suppression of ISG expression. We revealed a unique paradigm in viral immune evasion, in which EIAV co-opts the PKR-eIF2α axis to globally suppress interferon responses and facilitate viral replication. These findings therefore provide new insight into the pathogenesis of EIAV and might serve as a theoretical basis for clinical drug research and development.

## Materials and Methods

### Ethics statement

The use of horses in this study was approved by the Harbin Veterinary Research Institute (HVRI), the Chinese Academy of Agricultural Sciences (CAAS). All experiments adhered to the guidelines approved by the recommendations of the Ethics Committees of HVRI. The Animal Ethics Committee approval number is 231023–03-SW.

### Viruses and Cells

eMDMs were prepared from equine peripheral blood mononuclear cells as previously described and were maintained in RPMI 1640 (Sigma-Aldrich, R8758) supplemented with 30% newborn bovine serum (Ausbian, VS500N) and 30% horse serum (HyClone, SH30074.03). The human cell lines HEK293T and HeLa were maintained in Dulbecco’s modified Eagle’s medium (Sigma-Aldrich, D6429) supplemented with 10% fetal bovine serum (Alphabio, SA-201-1A) and 1% penicillin-streptomycin (Gibco, 15140122). The replication-competent EIAV strain EIAV_DLV36_ (stored in our laboratory) served as the model organism; its characteristics have been described previously [37].

### Antibodies and Reagents

The mouse anti-puromycin (MABE343), mouse anti-Flag (F1804), mouse anti-HA (H9658) monoclonal antibodies; the rabbit anti-Flag (F7425), rabbit anti-HA (H6908) antibodies and PKR inhibitor C16 (I9785) were purchased from Sigma-Aldrich. The rabbit anti-G3BP1 (ab181149), rabbit anti-PKR (ab184257), rabbit anti-phospho-PKR (ab32036), rabbit anti-phospho-GCN2 (ab75836) were purchased from Abcam. The mouse anti-G3BP1 (66486–1-Ig) monoclonal antibody; the rabbit anti-MDA5 (21775–1-AP), rabbit anti-IFITM3 (11714–1-AP), rabbit anti-ISG15 (15981–1-AP), rabbit anti-STAT1 (10144–2-AP) and rabbit anti-PERK (20582–1-AP) antibodies were purchased from Proteintech. The rabbit anti-phospho-eIF2α (AP0692), rabbit anti-GCN2 (A7155) and rabbit anti-β-actin (AC026) were purchased from Abclonal. The rabbit anti-eIF2α (TA6087S), rabbit anti-phospho-STAT1 (TP56498M) was purchased from Abmart.The rabbit anti-phospho-PERK antibody (3179S) was purchased from Cell Signaling Technology. The mouse anti-dsRNA J2 monoclonal antibody (A02181) was purchased from GeneScript. DyLight 680-labeled goat anti-rabbit (5230–0403) and DyLight 800-labeled goat anti-mouse (5230–0415) secondary antibodies were purchased from KPL. Monoclonal antibodies against P26 and Rev were prepared in our laboratory [52]. Lipofectamine LTX Reagent (Invitrogen, 15338100), Alexa Fluor 488-conjugated goat anti-mouse antibody (A32723) and Alexa Fluor 594-labeled Goat anti-Rabbit IgG (A11005) were purchased from Invitrogen. Equine IFN-α protein (RP0142E), equine IFN-β protein (RP0935E-005) and human IFN-α protein (RP1628H) were purchased from Kingfisher Biotech. Puromycin (A1113803) was purchased from Thermo Fisher Scientific.

### Plasmid construction

The pCMV3–8 vector expressing the full-length EIAV proviral genome was maintained in our lab [59]. Viral genes were amplified from pCMV3–8 and cloned into the pcDNA3.1 vector (Invitrogen, V79020) with a C-terminal 2 × HA tag. Equine G3BP1 was amplified from cDNA of eMDMs using RT-PCR and inserted into the pCMV-3 × Flag vector (Sigma-Aldrich, E7658). The pEGFP-N1 vector (HG-VYC0086) was purchased from Clontech. The pRL-TK vector (E2241) was purchased from Promega. The mutants of Rev and G3BP1 were generated using PCR-based site-directed mutagenesis or deletion mutagenesis. Transfection of plasmids was performed with Lipofectamine LTX Reagent (Invitrogen, 15338100) according to the manufacturer’s instructions.

### RT-qPCR

Total RNA was extracted from cells using an RNeasy mini kit (Qiagen, 74106) and reverse-transcribed with a PrimeScript RT reagent kit using a gDNA Eraser (Takara, RR047B). The mRNA levels were quantified using SYBR-Green (Takara, RR430A)-based real-time quantitative PCR analysis on QuantStudio 5 (Applied Biosystems), with β-actin as a control. The relative expression of each gene was calculated using the delta-delta -Ct method (2^-ΔΔCt^). Viral RNA was extracted from culture supernatants using a viral RNA extraction kit (Qiagen, 54161) and quantified by qPCR with Gag-specific primers, with a 10-fold dilution series of Gag standards used to generate standard curves.

### Western blot and Co-IP assay

Cells were lysed in ice-cold lysis buffer (50 mM Tris-HCl [Biosharp, 0234], pH 7.5, 50 mM NaCl [Sigma-Aldrich, S7653], 1% Triton X-100 [Sigma-Aldrich, T7878], 5 mM EDTA [Sigma-Aldrich, E6758]) containing 1% Protease Inhibitor Cocktail (MCE, HY-K0010). Proteins were separated by 4–20% SDS-PAGE and transferred to nitrocellulose membranes. After blocking with 5% fat-free milk (BD, 232100) in Tris-buffered saline for 2 h at room temperature (RT), membranes were probed with primary antibodies (4 °C overnight or RT 4 h) and secondary antibodies (RT 1 h).

For the Co-IP assay, the cellular extracts were untreated or were pre-treated with RNase A (100 μg/mL, Thermo Fisher, EN0531) at 37 °C for 30 min to assess RNA dependence, and were then incubated with 20 μL anti-Flag beads (Sigma-Aldrich, M8823) or anti-HA beads (MCE, HY-K0201) at 4 °C for 8 h. After washing with PBS, the beads were boiled in sample buffer and analyzed using SDS-PAGE and immunoblotting. Protein signals were detected and quantified using the Odyssey Imaging System (Li-Cor Biosciences). All immunoblot and Co-IP experiments were performed independently at least three times, with representative experiments being shown.

### Ribopuromycylation assay

This method labels actively translating polypeptides with puromycin, which can be detected using anti-puromycin antibody. Briefly, cells were pulsed with puromycin (5 µg/mL) for 30 min before lysis to capture ongoing translation. Subsequently, the cells were subjected to western blotting or immunofluorescence assay.

### Luciferase reporter assay

HEK293T cells seeded in 48-well plates were co-transfected with 10 ng of pRL-TK plasmid along with increasing concentrations (0, 10, 25, or 50 ng/well) of pcDNA3.1-Rev-HA or (0, 50, 100, or 200 ng/well) of pCMV3–8, with the total DNA amount adjusted to 210 ng/well using empty vector. At 24 hours post-transfection (hpt), the cell extracts were harvested in a passive buffer, and a luciferase reporter assay system (E1960, Promega) was used to determine the Renilla luciferase activities according to the manufacturer’s instructions.

### Confocal microscopy

Cells were fixed with 4% paraformaldehyde (15 min), permeabilized with 0.1% Triton X-100 (15 min), and blocked with 5% fat-free milk in PBS (1 h). Primary antibody incubation was performed (2 h RT or 4 °C overnight), followed by PBS washes and secondary antibody incubation (1 h). Nuclei were counterstained with DAPI (10 min). Images were acquired using a Zeiss LSM880 confocal microscope (Carl Zeiss), with signal quantification and colocalization analysis performed in Image J.

### Flow cytometry assay

HEK293T cells were seeded in 24-well plates and transfected with the indicated expression vectors. At 24 hpt, the cells were gently trypsinized and collected into 15 mL Falcon tubes. The cells were then pelleted by centrifugation, washed twice with ice-cold PBS, and resuspended in 500 µL of PBS. Fluorescence-activated cell sorting (FACS) analysis was conducted using a 488 nm excitation laser, and GFP fluorescence was detected in the FITC channel. A total of 10,000 cells per sample were analyzed, and the MFI of GFP was quantified. Data were analyzed using FlowJo software.

### Establishment of knockout cell lines

HEK293T cells were used to generate G3BP1 knockout cells using CRISPR-Cas9 technology. The gRNA was designed using the Broad Institute Zhang Lab Guide Design Resources. The selected gRNA sequence was: 5’-GCTCATGCCACGCTAAATGATGG-3’. A synthetic DNA fragment encompassing the G3BP1-targeting gRNA, along with a gRNA scaffold, a U6 promoter, and a U6 terminator, was synthesized and inserted into the pMD18-T vector (Clontech, 6011). The Cas9-eGFP expression construct (pMJ920) was a generous gift from Dr. Jennifer Doudna (University of California, Berkeley). For knockout generation, HEK293T cells cultured in 6-well plates were transfected with 1.0 μg each of the G3BP1 gRNA plasmid and the pMJ920 plasmid. At 36 hpt, GFP-positive cells were isolated by FACS. Single-cell-derived clones were subsequently expanded, and G3BP1 knockout was validated by western blotting.

### Oligo(dT) FISH

A CY3-labeled oligo-dT (25) probe was synthesized by Genepharma. Oligo(dT) FISH was performed using the Small RNA FISH Kit (Genepharma, F40261) according to the manufacturer’s instructions. HeLa cells transfected with the indicated plasmids were fixed with 4% formaldehyde, permeabilized with 0.1% Buffer A (Triton X-100), and incubated with 2 × Buffer C (2 × saline-sodium citrate [SSC]) at 37°C for 30 min. The CY3-labeled oligo-dT(25) probe was diluted in Buffer E (hybridization buffer containing dextran sulfate, SSC, formamide, tRNA, and BSA), denatured at 73°C for 5 min, and hybridized at 37°C overnight. After washing with 0.1% Buffer F (Tween 20 in 4 × SSC) and Buffer C, immunostaining was then performed as described above. Images were acquired using a Zeiss LSM880 confocal microscope, and colocalization analysis was performed using ImageJ.

### RNA interference

eMDMs were seeded into 6-well plates and cultivated for 36 h and then were transfected with equine G3BP1 or scrambled siRNA with Lipofectamine RNAiMAX (Invitrogen, 13778075). The efficiency of siRNA silencing of G3BP1 was evaluated using western blotting. The targeting sequence for the equine G3BP1 gene is GGAGAUUCAUGCAGACAUU.

### Statistical analysis

All experiments were repeated independently at least three times. All the graphs and relevant analyses were conducted using GraphPad Prism. Statistical significance between two groups was analyzed using two-tailed unpaired Student’s t-tests. Error bars indicate means ± standard deviations (SD). NS, not significant (*P* > 0.05), *P < 0.05, **P < 0.01, ***P < 0.001, ****P < 0.0001.

## Supporting information

S1 Fig **EIAV infection attenuates ISG protein induction in response to IFN-β**.
(**A-C**) mRNA levels of MDA5, IFITM3, and ISG15 in uninfected and EIAV-infected eMDMs. eMDMs were either mock-infected or infected with EIAV (MOI = 1) for 12 or 24 hours, and then treated with either 100 U/mL IFN-β or mock-treated with PBS for an additional 12 hours. Cellular RNA was extracted, and the levels of MDA5 (A), IFITM3 (B), and ISG15 (C) mRNA were determined using RT-qPCR. β-actin mRNA quantification from the same samples was used for normalization. Data represent means ± SD of three independent experiments. (**D**) Protein levels of MDA5, IFITM3, and ISG15 in uninfected and EIAV-infected eMDMs. Western blot analysis was performed to examine the protein expression of STAT1, p-STAT1, MDA5, IFITM3, and ISG15 in the samples corresponding to Fig S1A. β-actin was used as a loading control, and EIAV infection was verified using an anti-P26 antibody. The intensities of the protein bands were analyzed using the Odyssey Imaging System to calculate values relative to those of β-actin. Results were normalized to IFN-β-treated mock cells (24h) as the control group, which was set to 1.
(TIF)

S2 Fig **EIAV infection induces a global shutoff of host protein synthesis**.
(**A**) Dose-dependent suppression of global protein synthesis resulting from EIAV proviral DNA expression. HEK293T cells were seeded into 6-well plates and transfected with increasing amounts (250, 500 and 1000 ng) of pCMV3–8 using Lipofectamine LTX reagent. Empty vector served as negative control. At 24 hpt, cells were pulsed with 5 µg/mL puromycin for 30 min to label nascent polypeptides. Whole cell lysates were analyzed using western blotting with the indicated antibodies. (**B**) Dose-dependent suppression of Renilla luciferase activity resulting from EIAV proviral DNA expression. HEK293T cells were seeded into 48-well plates and co-transfected with pRL-TK reporter plasmid (10 ng) and increasing amounts (50, 100, and 200 ng) of pCMV3–8. At 24 hpt, cells were lysed and subjected to Renilla luciferase assays. Data represent means ± SD of three independent experiments. (**C**) Overexpression of EIAV proviral DNA does not reduce Renilla luciferase mRNA levels. RT-qPCR analysis was performed to examine the mRNA levels of Renilla luciferase and β-actin in the samples corresponding to Fig S2B. Data represent means ± SD of three independent experiments. (**D**) Overexpression of EIAV proviral DNA reduces EGFP fluorescence intensity. HeLa cells were co-transfected with pEGFP-N1 (200 ng) and either pCMV 3–8 (500 ng) or with empty vector. Confocal microscopy was used to visualize EGFP fluorescence at 24 hpt. EIAV Gag was detected by indirect immunofluorescence (red), and DAPI staining (blue) was performed to visualize nuclei. Scale bars: 10 µm. (**E**) Quantification of the relative EGFP fluorescence intensity in Fig S2D. Data represent means ± SD of three independent experiments. (**F**) Overexpression of EIAV proviral DNA does not reduce EGFP mRNA levels. RT-qPCR assays were conducted to determine the mRNA levels of EGFP and β-actin in the samples corresponding to Fig S2E. Data represent means ± SD of three independent experiments. (**G**) Flow cytometric analysis of EGFP fluorescence in HEK293T cells expressing EIAV proviral DNA. HEK293T cells were co-transfected with pEGFP-N1 (200 ng) and either pCMV3–8 (500 ng) or empty vector. At 24 hpt, cells were harvested and subjected to flow cytometric analysis to quantify the MFI of EGFP. Data are presented as means ± SD from three independent experiments. (**H**) Representative flow cytometry histograms showing EGFP fluorescence intensity corresponding to (G).
(TIF)

S3 Fig **EIAV Rev induces a global shutoff of host protein synthesis**.
(**A**) Dose-dependent activation of the PKR-eIF2α pathway by EIAV Rev. HEK293T cells were seeded into 6-well plates and transfected with increasing concentrations of pcDNA 3.1-Rev-HA (50,100, and 200 ng) or with empty vector. Protein expression was analyzed using western blotting with the indicated antibodies at 24 hpt. (**B**) Dose-dependent suppression of Renilla luciferase activity resulting from EIAV Rev expression. HEK293T cells were seeded into 48-well plates co-transfected with pRL-TK reporter plasmid (10 ng) and increasing amounts (10, 25, and 50ng) of pcDNA3.1-Rev-HA or with empty vector. At 24 hpt, cells were lysed and subjected to Renilla luciferase assays. Data represent means ± SD of three independent experiments. (**C**) Overexpression of EIAV Rev does not reduce Renilla luciferase mRNA levels. RT-qPCR analysis was performed to examine the Renilla luciferase and β-actin mRNA levels in the samples corresponding to Fig S3B. Data represent means ± SD of three independent experiments. (**D**) EIAV Rev reduces global protein synthesis. HeLa cells transfected with the pcDNA 3.1-HA or pcDNA3.1-Rev-HA (500 ng) for 24 h were incubated with 5 µg/mL puromycin at 37 °C for 30 min. The cells were then fixed, permeabilized, and processed for immunofluorescence analysis. An HA-specific monoclonal antibody was used to detect Rev (red). Puromycylated chains are visualized using an anti-puromycin antibody (green). Nuclei were stained with DAPI (blue). Scale bars: 10 µm. Shown is an intensity profile of the linear ROI across the HeLa co-stained with puromycin and Rev.
(TIF)

S4 Fig **Oligo(dT) FISH analysis of mRNA distribution**.
(**A**) HeLa cells were transfected with pcDNA3.1-Rev-HA, pCMV3–8, or pcDNA3.1-HA (500 ng). At 16 hpt, cells were treated with 1 μM C16 or DMSO for an additional 8 h. Cells were fixed and subjected to oligo(dT) FISH to detect poly(A) mRNA (red) and immunostaining to detect Rev protein (green). Nuclei were counterstained with DAPI (blue). Scale bars: 10 µm. Shown is an intensity profile of the linear ROI across the HeLa cell subjected to FISH for poly(A) mRNA and immunostaining for Rev protein.
(TIF)

S5 Fig **Effect of G3BP1 overexpression on Rev-mediated PKR-eIF2α activation**.
(**A**) Immunoblot analysis of PKR/eIF2α phosphorylation and puromycin incorporation in cells expressing Rev together with wild-type G3BP1 or G3BP1-ΔNTF2L. HEK293T cells were co-transfected with pcDNA3.1-Rev-HA (500 ng) together with either wild-type G3BP1 (500 ng), G3BP1-ΔNTF2L (500 ng), or empty vector (500 ng). At 24 hpt, cells were pulsed with 5 µg/mL puromycin for 30 min prior to harvest. Protein expression was analyzed using western blotting with the indicated antibodies. Results were normalized to cells transfected with wild-type Rev.
(TIF)

S6 Fig **HIV-1 Rev does not induce stress granule formation**.
(**A**-**B**) Co-IP analysis of the interaction between HIV-1 Rev and G3BP1. HEK293T cells were co-transfected with G3BP1-Flag (500 ng) together with either HIV-1 Rev-HA (500 ng) or EIAV Rev-HA (500 ng). At 24 hpt, cell lysates were subjected to immunoprecipitation using anti-HA (A) or anti-Flag (B) beads, followed by western blotting with the indicated antibodies. (**C**) Immunofluorescence analysis of G3BP1 colocalization with HIV-1 Rev. HeLa cells were transfected with EIAV Rev-HA or HIV-1 Rev-HA. At 24 hpt, cells were fixed and stained with anti-HA (red) and anti-G3BP1 (green) antibodies. Nuclei were counterstained with DAPI (blue). Scale bars: 10 µm. Shown is an intensity profile of the ROI across the HeLa co-stained with G3BP1 and Rev. (**D**) Quantification of the SG-positive cells in Fig S6C.
(TIF)

S7 Fig **Effect of Rev mutants on host protein synthesis and mRNA distribution**.
(**A**) Oligo(dT) FISH analysis of mRNA distribution in cells expressing Rev or its mutants. HeLa cells were transfected with pcDNA3.1-Rev-HA (wild-type) or its mutants. At 24 hpt, cells were fixed and subjected to oligo(dT) FISH to detect poly(A) mRNA (red) and immunostaining to detect Rev protein (green). Nuclei were counterstained with DAPI (blue). Representative images show the distribution of mRNA and Rev in transfected cells. Scale bars: 10 µm. Shown is an intensity profile of the ROI across the HeLa cell subjected to FISH for poly(A) mRNA (red) and immunostaining for Rev protein (green). (**B**) Immunofluorescence analysis of puromycin incorporation in cells expressing Rev or its mutants. HeLa cells were transfected with pcDNA3.1-Rev-HA (wild-type) or its mutants (500 ng). At 24 hpt, cells were pulsed with 5 µg/mL puromycin for 30 min prior to fixation. Puromycin-labeled nascent polypeptides were detected by immunostaining (green), and Rev protein was detected by immunostaining (red). Nuclei were counterstained with DAPI (blue). Representative images are shown. Scale bars: 10 µm. Shown is an intensity profile of the ROI across the HeLa co-stained with puromycin and Rev.
(TIF)

S1 Table **Oligo sequences used for qPCR**.
(DOCX)
