# Supplementary material for: Equine infectious anemia virus blocks interferon responses through Rev-mediated activation of the stress granule-PKR-eIF2α pathway
Source: PLoS Pathog. 2026 May 21;22(5):e1014262. doi: 10.1371/journal.ppat.1014262 (PMC13221151; doi:10.1371/journal.ppat.1014262)
Supplement: S1 Table — Oligo sequences used for qPCR. (DOCX) [file ppat.1014262.s008.docx]

| Name | Sequences (5’→3’) |
| --- | --- |
| β-actin-F | ACGGCATCGTCACCAACTG |
| β-actin-R | CAAACATGATCTGGGTCATCTTCTC |
| equine MDA5-F | TACCTCAAATACTGGGACT |
| equine MDA5-R | CATCAAGATTGGCACATA |
| equine IFITM3-F | GGGCTTCGTGGCTTTC |
| equine IFITM3-R | CAGATGTTCAGGCACTTGG |
| equine ISG15-F | CCGCAGCAGTGCCTATGA |
| equine ISG15-R | ACCCGGCTGTAAGCTCGTA |
| Rluc-F | ATAACTGGTCCGCAGTGGTG |
| Rluc-R | TAAGAAGAGGCCGCGTTACC |
| EGFP-F | AAGGGCATCGACTTCAAGGA |
| EGFP-R | CTTCTCGTTGGGGTCTTGC |
| Gag-F | CGATGCCAAATCCTCCATTAG |
| Gag-R | CTGATCAAAAGCAGGTTCCATCT |
| human MDA5-F | ACGAGAATTTCCGCTATCTC |
| human MDA5-R | CCACGAATTCCCGAGTCCAA |
| human IFITM3-F | TCAAGGAGGAGCACGAGG |
| human IFITM3-R | AACAGGGACCAGACGACAT |
| human ISG15-F | GACAAATGCGACGAACCTCT |
| human ISG15-R | CGGCCCTTGTTATTCCTCA |

**S1Table . Oligo sequences used for qPCR**
